# Dexmedetomidine inhibits unstable motor network in patients with primary motor area gliomas

**DOI:** 10.18632/aging.203077

**Published:** 2021-05-25

**Authors:** Tao Yu, Songlin Yu, Zhentao Zuo, Nan Lin, Jing Wang, Yuanli Zhao, Song Lin

**Affiliations:** 1 Department of Neurosurgery, Beijing Tiantan Hospital, Capital Medical University, Beijing 100070, China; 2 China National Clinical Research Center for Neurological Diseases, Beijing 100070, China; 3 State Key Laboratory of Brain and Cognitive Science, Institute of Biophysics, Chinese Academy of Sciences, Beijing 100101, China; 4 University of Chinese Academy of Sciences, Beijing 100049, China; 5 CAS Center for Excellence in Brain Science and Intelligence Technology, Chinese Academy of Sciences, Shanghai 200031, China; 6 Department of Anesthesiology, Beijing Tiantan Hospital, Capital Medical University, Beijing 100070, China; 7 Department of Neurosurgery, Peking University International Hospital, Peking University Health Science Center, Beijing 102206, China; 8 Department of Neurosurgery, Beijing Neurosurgical Institute, Capital Medical University, Beijing 100070, China

**Keywords:** network reorganization, functional magnetic resonance imaging, dexmedetomidine, motor network, glioma

## Abstract

Background: Sedative agents such as dexmedetomidine have been found to transiently exacerbate or unmask limb motor dysfunction in patients with eloquent area brain gliomas. The present study aims to investigate whether dexmedetomidine can inhibit motor plasticity in patients with glioma via fMRI.

Methods: 21 patients with brain glioma were prospectively recruited between September 2017 and December 2018. Patients were classified into pre-M1 (primary motor cortex) group (n=9), post-M1 group (n=6), and non-eloquent group (control group) (n=6) according to the tumor position related to M1. The hand movement task-fMRI and resting state fMRI (rs-fMRI) were performed before and after sedation using dexmedetomidine. The lateralization index (LI) of activation voxels and magnitude and the functional connectivity (FC) of motor network were compared before and after sedation and among different groups.

Results: Permanent postoperative motor deficit of the upper limb was found in 5 of 6 patients in the pre-M1 group, and none in other groups (*P* < .01). Task-fMRI showed the LI of activation volume and activation magnitude at M1 significantly increased only in the pre-M1 group after sedation (*P* < .05). Rs-fMRI showed 60.0% (27 of 45) FCs of motor network decreased in pre-M1 group after sedation (p[FDR] < .05); whereas there was no FC reduction in post-M1 and control groups (p[FDR] > .05).

Conclusions: In patients with eloquent area gliomas, dexmedetomidine can inhibit the unstable compensative motor plasticity on both task- and rs-fMRI. fMRI may be a promising method for elucidating the effect of sedative agents on motor plasticity.

## INTRODUCTION

For patients with tumors near eloquent regions, repeated neurologic function assessments are needed after surgery to monitor neurologic performance. These patients often show transient contralesional limb weakness when they were sedated in the operating room, the postanesthesia care unit, and the intensive care unit [1, 2]. Lin et al. reported transient limb weakness caused by dexmedetomidine in 23% patients with frontal-parietal tumors [1]. It was supposed that motor plasticity was temporarily suppressed by the sedation drugs, and thus the limb dysfunction was unmasked or exacerbated [1, 2], but validation evidence is lacking [3].

It is well known that anesthetic agents widespread decrease brain metabolism and cerebral activation [4, 5]. Dexmedetomidine has been proved to cause a significant drop in the capacity for efficient information transmission at both the local and global levels [6]. Furthermore, dexmedetomidine doesn’t suppress FCs of the motor network in a healthy volunteer [5], but its influence on the motor network in the pathological state remains uninvestigated. As a commonly used sedative drug, dexmedetomidine is a highly selective agonist of α2- adrenoceptors and can inhibit the secretion of norepinephrine [7], which is an important neurotransmitter in the process of functional remodeling [8]. We suppose dexmedetomidine can induce the instability of motor network if tumors significantly invade the motor area.

Brain tumors can lead to brain motor function remodeling or functional plasticity to maintain motor function, especially for those invading primary motor cortex (M1) [9, 10]. The task-based functional MRI (task-fMRI) has been a useful tool for locating the critical functional areas and grading the extent of motor plasticity [11, 12]. Resting-state fMRI (rs-fMRI) was also used to locate functional areas and evaluate motor execution networks [13–15]. For patients with gliomas involving motor areas, the activation areas on task-fMRI and FCs within motor network can reflect the local compensations and remote recruitments [16, 17]. Thus, we use fMRI as a tool and try to find out whether motor plasticity is influenced by sedation in these patients.

In this study, patients with motor eloquent glioma were divided into different groups according to tumor locations relative to the rolandic area. We presume that pre-central gyrus glioma causes the most significant and unstable motor plasticity. Patients were sedated using dexmedetomidine and underwent task- and rs-fMRI before and after sedation. We aimed to investigate whether the motor plasticity is unstable and can be altered by dexmedetomidine and try to explain why the patient experiences transient limb weakness after sedation.

## MATERIALS AND METHODS

### Participants characteristics

The study prospectively recruited 21 patients (12 males, age = 42±12 (mean±SD) years) who were diagnosed as brain frontal-parietal gliomas between September 2017 and December 2018. The study was approved by the appropriate Institutional Review Board (IRB), and written informed consent was obtained from all subjects. Sixteen patients received surgical treatments. Inclusion criterion: patients with supratentorial mass lesion; aged 18 to 75; ASA I-II; MRI compatible. Exclusion criteria:unable to comprehend and cooperate with the behavioral and fMRI examination; impaired mental status; taking sedative drugs in the past 24 hours; taking pain reliever in the past 24 hours; drug and/or alcohol abuse; pregnant and/or lactation period woman. All patients accomplished the task-fMRI and resting-state fMRI (rs-fMRI) examinations. The patients were divided into three groups according to the spatial location of tumor and M1: the tumor was located in SMA, PMd and M1 (pre-M1 group, 9 patients), in postcentral gyrus (post-M1 group, 6 patients) and midline postcentral gyrus (control group, 6 patients).

### The work-flow of experiment and sedation protocol

**Figure 1:**
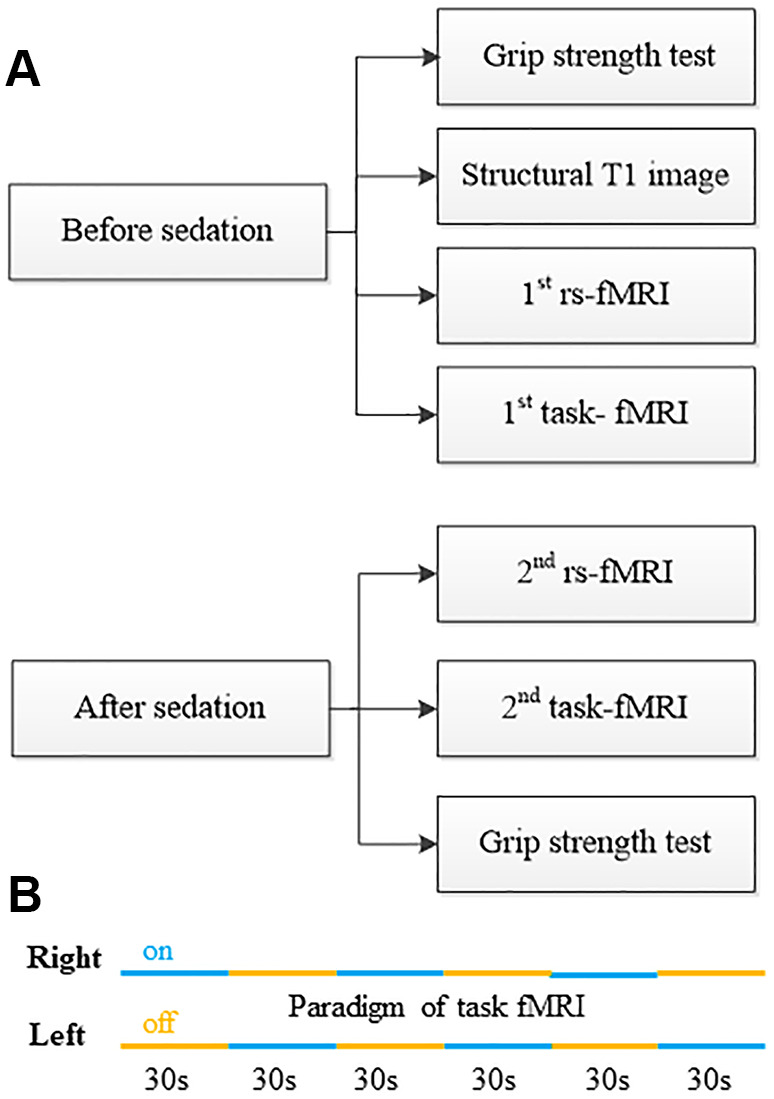
**Work flow of physiological test, fMRI data acquisition and sedation protocol.** (**A**) Study work flow. (**B**) Paradigm of task-fMRI.

The work-flow of experiment before and after sedation is showed in Figure 1A. Subjects fasted for at least 6h from solids and 2h from liquids before sedation. During the study and the recovery period, an anesthesiologist monitored electrocardiogram, blood pressure, pulse oximetry (SpO_2_), and breathing frequency (Monitor: MAGLIFE C PLUS; Schiller Medical; Germany). After the first several tests, dexmedetomidine (Jiangsu Hengrui Medicine Co., Ltd., China) was infused through an intravenous catheter placed into a vein of the right hand or forearm. Dexmedetomidine was administered as a 1μg/kg loading bolus over 10min, followed by a 0.7μg/kg/h infusion. The sedation states were judged by the anesthesiologist who adjusted the drug administration as the study needed. The patients’ sedation level was evaluated by Observer’s Assessment of Alertness/Sedation (OAA/S) scale that was developed to measure the level of alertness in subjects who are sedated [18]. The OAA/S is scored from 1 to 5, indicating deep sleep to fully alert (5 = alert, 4 = lethargic, 3 = aroused by voice, 2 = aroused by shaking, 1 = deep sleep). The titrated sedative doses were guided by OAA/S, targeting a score ≤ 3. Once achieved a score of 3, the second rs-fMRI and task-fMRI was performed. At last the second muscle power test was performed and then the drug infusion was stopped. Throughout the study, the subjects breathed spontaneously. The vital signs were stable, and no study was interrupted because of the drug administration.

### fMRI data acquisition

MRI data were performed at a Siemens Verio 3.0 Tesla MRI scanner (Siemens Healthineers, Erlangen, Germany). A three-plane localizer image was initially obtained to identify anatomic landmarks and to allow positioning of the transverse sections parallel to the anterior commissure–posterior commissure line. The head of each participant was snugly fixed by foam pads to reduce head movements and scanner noise. The structural images were obtained in a sagittal orientation employing a magnetization prepared rapid gradient echo (MPRAGE) sequence over the whole brain: 192 slices, TI/TR/TE = 900/2,300/3.25msec, flip angle: 8°, slice thickness 1.0 mm, field of view = 256 × 256 mm^2^, matrix = 256 × 256.

The brain task-fMRI (Figure 1B) was performed during alternating left and right four finger-to-thumb opposition movements by using T2*-weighted blood oxygen level-dependent based Echo-planar MR imaging (36 slices, repetition time/echo time = 3,000/30 ms, flip angle = 90°, field of view = 192 × 192 mm^2^, matrix=64 × 64, slice thickness 3.0 mm, 0.75mm intersection gap, in plane resolution: 3mm × 3mm). The task instructions were auditory-cued using a digital audiotape. All subjects were visually monitored during the experiment to ensure compliance with the protocol. After drug was infused, if the patient stopped moving because of drowsiness, he/she would be verbally reminded to continue immediately through speaker. If there were more than 2 stops in one task block, the task would be aborted and repeated.

Rs-fMRI was also performed before and after dexmedetomidine was administered, with blood oxygen level–dependent (BOLD) gradient-echo T2*-weighted echo-planar imaging (32 slices, repetition time/echo time = 2,220/30 ms, flip angle = 90°, field of view = 192 × 192 mm^2^, matrix= 64 × 64, slice thickness 3 mm, 1mm intersection gap, in plane resolution: 3mm × 3mm). During the echo-planar imaging data acquisition, subjects were instructed to keep relaxed with their eyes closed and remain motionless as much as possible. Each scan lasted for 9 min 6 secs and 246 volumes were obtained.

### Regions-of-interest (ROI) creation in the motor network of rs-fMRI

Ten ROIs were selected to detect the functional connectivity (FC) of the hand motor network. We focused on the dynamic changes in the organization of the motor execution network controlling for the movement of the contralesional hand. First, because the M1 may be damaged or significantly shifted from the original site, we define the ROI of M1 individually. We used the voxels of task-evoked activation to create an individual mask of M1, and then the mask was normalized using the non-linear transformation to MNI space. For supplementary motor area (SMA), because in all cases there was activation during task-fMRI, for the sake of simplicity we used an individual spherical region (radius = 5.0 mm) centered at the peak activation point of SMA. The ipsilesional dorsal premotor area (PMd) was damaged in pre-M1 group, so in all cases we only used contralesional PMd spherical ROI with 5.0 mm radius and defined it in MNI space (left -22, -13, 57; right 28, -10, 54) [15, 19]. For other ROIs, we also used MNI coordinates from previous reports including bilateral thalamus (ventral lateral nucleus), superior cerebellum (lobule VI), and dentate nucleus (Table 1) [19].

**Table 1:** Common sites of ROIs constituting hand motor execution network (Radius: 5mm)*.

**Region of interest**		**Abbreviation**	**MNI coordinates**		
			*X*	*Y*	*Z*
Superior cerebellum (lobule VI)	L	SCb	-18	-54	-22
	R		16	-52	-22
Dentate nucleus	L	Den	-28	-55	-33
	R		19	-55	-30
Thalamus (Ventral Lateral)	L	Th	-11	-15	8
	R		13	-15	8

*The ROIs of bilateral M1, SMA, PMd were constructed individually, which was described in detail in the text.

### Data pre-processing and analysis of task-fMRI and rs-fMRI

The task-fMRI images were preprocessed using DPABI standard pipeline including slice timing, realignment, structural images co-registration to functional images, segment (DARTEL) and smoothing [20]. Statistical analyses of functional images were performed in MATLAB 2015b (Mathworks, Natick, MA, USA) with SPM12 (Wellcome Centre of Human Neuroimaging, London, UK) using the general linear model. For each subject, images were corrected for subject motion with the first volume of each study used as a reference. Volumes affected by excessive motion (10 mm displacements) were discarded. The EPI images were co-registered with anatomic images. Tumor segmentation was performed based on the high resolution T1 anatomical dataset. The resulting images were smoothed with a Gaussian spatial filter to a final smoothness of 5 mm. The results of individual analyses were thresholded at the *p* < 0.05 level of significance corrected for family wise error (FWE). For less conservative assessment, if there was no activation or cluster size < 10, the uncorrected threshold of *p* < 0.001 was used. The functional image was then overlaid on a high resolution T1 structural image in order to have the anatomic localization of the functional foci.

The rs-fMRI data were also preprocessed using DPABI standard pipeline. The first 10 volumes were discarded to allow for magnetization equilibrium effects and the adaptation of the subjects to the circumstances, leaving 236 volumes for further analysis. The processing steps included slice timing, realignment and structural images co-registered to functional images. Structural images were normalized with new segment and DARTEL. The nuisance covariates were regressed out with Friston 24 (6 head motion parameters), CSF and white matter. Then the functional images were normalized, detrended, smoothed (Gaussian kernel full width half maximum = 4 mm), and temporal band pass filter (0.01–0.1 Hz) was used to reduce low-frequency drift and high-frequency physiological noise. Rs-fMRI time courses of each ROI were extracted, and ROI-to-ROI FC values were calculated using Pearson correlation and transformed to Fisher z.

### Maximum grip strength test

The maximum grip strength was measured before and after sedation using a digital dynamometer (Zhongshan Camry Electronic Co. Ltd, Guangdong, China) as motor ability. Lateralization index (LI) of maximum grip strength was defined as (C–I)/(C+I) where C and I mean the contralateral and ipsilateral maximum grip strength.

### Surgery strategy and clinical results

Participant sociodemographic and medical variables (Table 2) were archived in a purpose-made database for later analysis. Surgery was performed by one senior neurosurgeon with intraoperative neurophysiological monitoring (appendix). All the participants were evaluated on preoperative day 1, postoperative day 7, and at follow-up after 1 month regarding their motor function.

**Table 2:** Demographic data and surgical results of the 21 patients.

**Characteristics**	**Pre-M1 group**	**Post-M1 group**	**Control group**	**Total**	***p***
Number of patients	9	6	6	21	
Age (years, Mean±SD)	44±12	38±13	43±13	42±12	
Sex (Male/Female)	5/4	2/4	5/1	12/9	
Tumour Volume (mean, cm^3^)	33.4±26.9	36.0±15.5	9.5±5.7	27.3±22.2	
Tumour invasion (mainly)					
postcentral gyrus or midline	0	6	6	12	***
SMA or PMA	9	0	0	9	***
precentral gyrus	9	0	0	9	***
Tumour side (L/R)	5/4	3/3	2/4	10/11	
Surgery	6	5	5	16	
Extent of resection					
Total resection	4	3	4	11	
Subtotal resection	2	2	1	5	
Partial resection	0	0	0	0	
Pathological report					
WHO I glioma	1	0	0	1	
WHO II glioma	2	2	2	6	
WHO III glioma	0	1	2	3	
WHO IV glioma	3	2	1	6	
Post-op muscle power^a^					
V	1	5	5	11	**
IV	3	0	0	3	
III	2	0	0	2	
Follow-up^b^					
muscle power improvement	2	0	0	2	
Recurrence or progression	1	0	0	1	
Mortality	0	0	0	0	

**^a^**indicates muscle power of upper limb.

**^b^**Follow-up time ranged from 1 to 10 months (mean, 6.8 months).

***p < 0.001.

**p < 0.01.

### Statistical analysis

Lateralization index (LI) of M1 was defined as (C–I)/(C+I) where C and I mean the contralateral and ipsilateral sensorimotor area to the ipsilesional hemisphere. Magnitude LI (LI-M) using the maximum T-value and volumetric LI (LI-V) using the number of activated voxels within the sensorimotor area was calculated. Statistical analyses were conducted using SPSS (IBM Corp., Chicago, IL, USA). The significance level was set at *P* < .05. The false discovery rate (FDR) correction was applied to control false positives from multiple comparisons. FDR adjusted *P* value (p[FDR]) less than 0.05 was considered significant.

## RESULTS

### Clinical characteristics and grouping of patients

**Figure 2:**
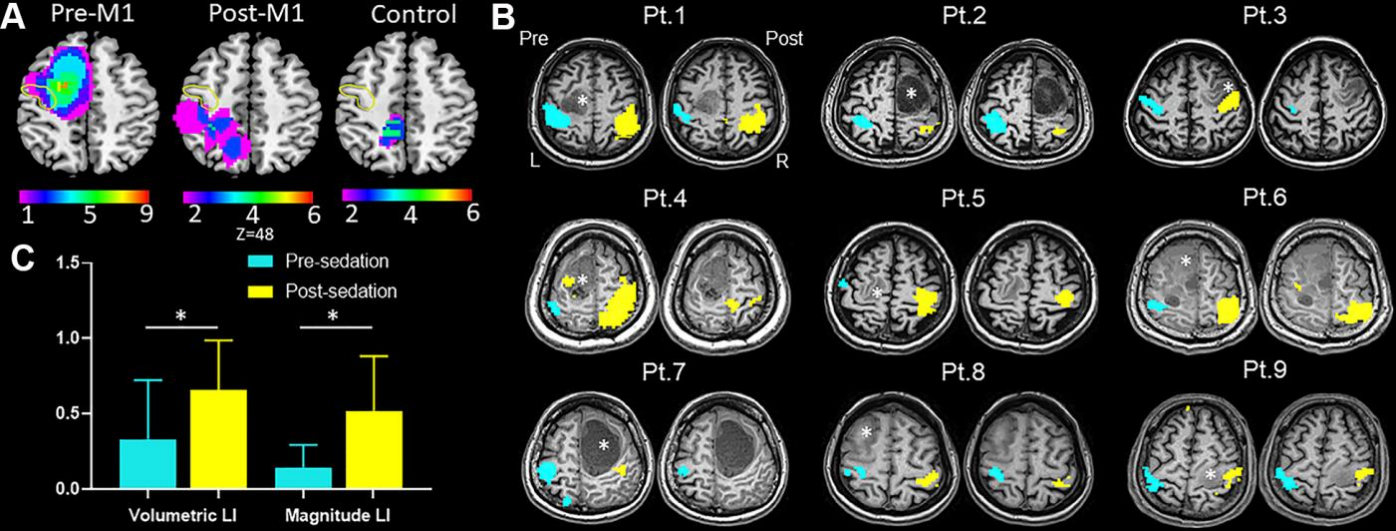
**Results of task-fMRI in M1 group.** (**A**) Probability maps of lesion distribution for M1, post-M1 and control groups. The yellow circle denoted location of anterior central gyrus. (**B**) Hand motor task-fMRI results of nine patients from M1 group. Axial individual anatomical images with superimposed functional activation pre- and post-administration of dexmedetomidine were presented. In the lesional hemisphere, activation of the hand task decreased significantly after sedation. Right (R) and left (L) hemispheres are marked. *indicates locations of the lesions. (**C**) The bar graph showed both magnitude lateralization index (LI-M) and volumetric LI (LI-V) of M1 increased significantly after sedation in M1 group (mean with 95% CI) (*P < .05).

The imaging and clinical data of the 21 patients are summarized in Table 2. All the patients underwent fMRI examinations before and after sedation successfully without any observed side effects. 16 patients received surgical treatments. After the operation, new motor deficits of upper-limb occurred in five of six patients in the pre-M1 group, and none in other groups (*P* < .01). Probability maps of lesion distribution of the three groups were shown in Figure 2A.

### Behavioral test

We found no significant difference of the motion counts among the three groups and no significant difference of the motion counts or maximum grip strength laterality between pre- and post-administration of dexmedetomidine within each group.

### Task-fMRI: sedation alters lateralization of hand motor areas

Figure 2B showed typical activation maps before and after sedation. In pre-M1 group, we found no statistical significance of either activation volume or activation magnitude between the lesional and healthy hemispheres before sedation; however, after sedation, both activation volume (*P* < .02) and activation magnitude (*P* < .02) in the lesional hemisphere was significantly lower than that in the healthy hemisphere, which were not observed in other groups. The LI-M and LI-V of M1 were strongly correlated both before (r = 0.901; *P* < .001) and after sedation (r = 0.908; *P* < .001). Both LI-V (*P* < .03) and LI-M (*P* < .02) increased significantly after sedation in pre-M1 group (Figure 2C). Surprisingly, in post-M1 group and control group, we found no significant differences for either LI-V (*P* = .173 versus .173) or LI-M (*P* = .344 versus .917) after sedation.

### Rs-fMRI: sedation alters FCs within the motor network

**Figure 3:**
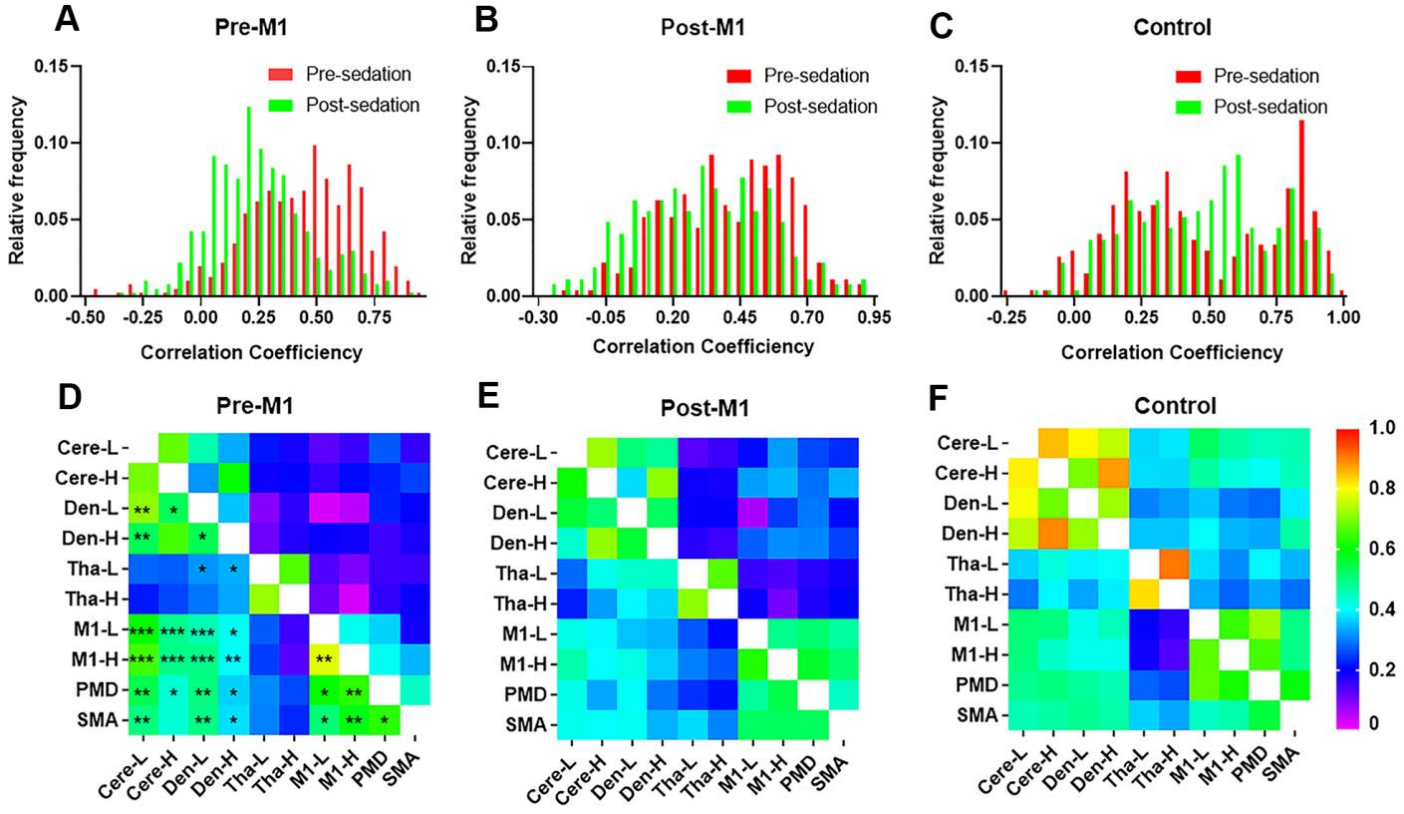
**The stability and distribution of functional connectivity (FC) within motor network.** Distribution of individual FCs in M1 group (**A**) and post-M1 group (**B**) shifted to the lower FCs after sedation (*p* < 0.0001), which was not observed in control group (**C**). (**D**–**F**) FCs within brain motor networks before (lower part of each matrix) and after (upper part of each matrix) sedation in three groups. (**D**) Most FCs within motor networks decreased significantly after sedation in M1 group (p[FDR] < 0.05) which was not observed in either post-M1 group (**E**) or control group (**F**) (* *P* < .05, ** *P* < .01, *** *P* < .001).

Before sedation, the FCs distribution of post-M1 (mean, 0.41; 95%CI, 0.39-0.44) were lower than that of pre-M1 group (mean, 0.44; 95%CI, 0.42-0.47; *P* < .05) and control group (mean, 0.47; 95%CI, 0.44-0.51; *P* < .05); however, we found no significant difference of FCs between pre-M1 group and control group (*P* = .376). After sedation, FCs distribution of pre-M1 group (mean 0.24; 95%CI, 0.22-0.26) was significantly lower than that of post-M1 (mean 0.31, 95%CI, 0.28-0.34, *P* < .0001) and control group (mean 0.47, 95%CI, 0.44-0.50, *P* < .0001); FCs distribution of post-M1 group were significantly lower than that of control group (*P* < .0001). Distribution of FCs in pre-M1 group (*P* < .0001) and post-M1 group (*P* < .0001) shifted to the lower FCs obviously after sedation (Figure 3A, 3B), which was not observed in control group (Figure 3C*, P* = 0.90).

**Figure 4:**
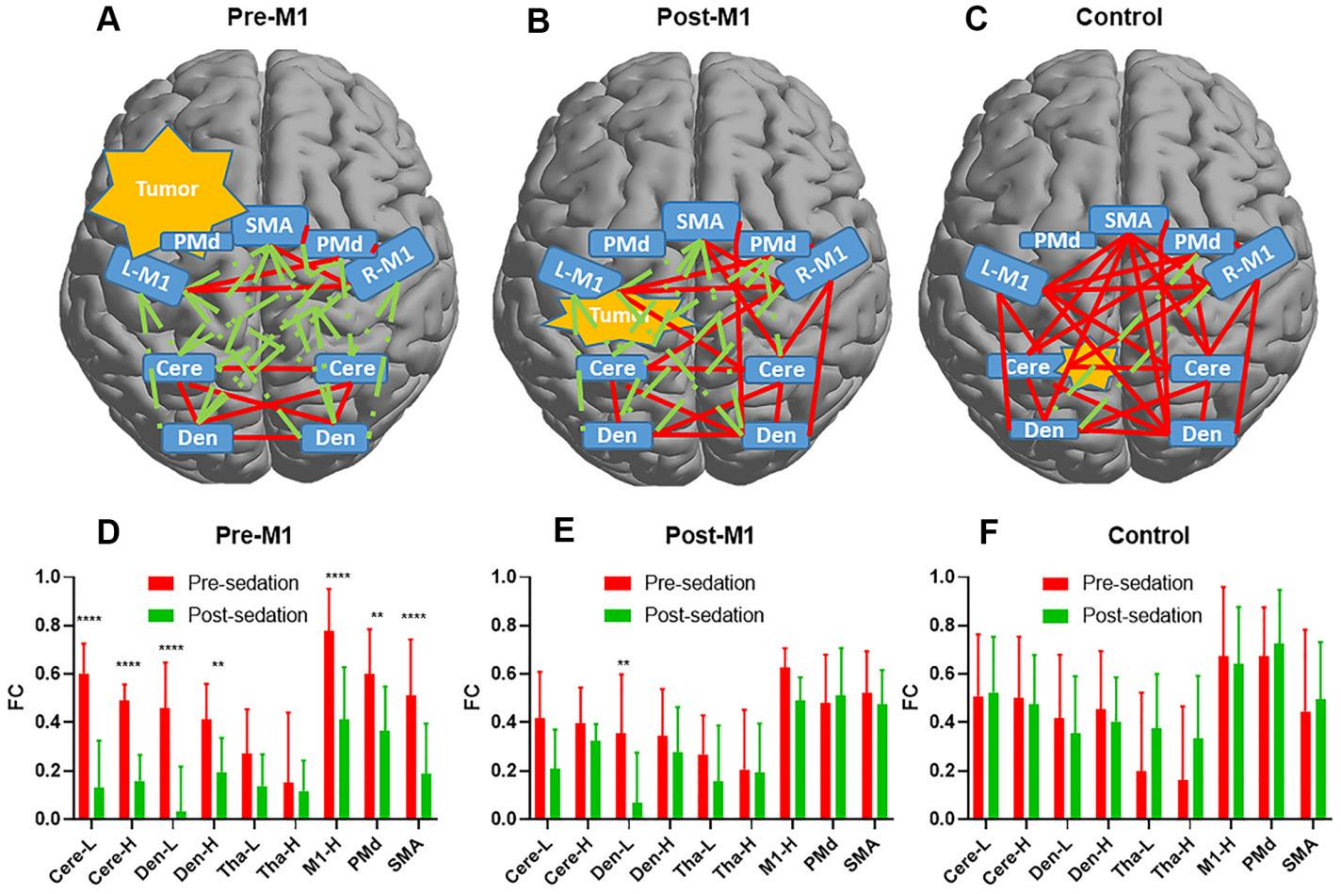
**The functional connectivity (FC) alteration before and after sedation.** Delineation of FCs after sedation for pre-M1 (**A**) post-M1 (**B**) and control (**C**) groups. Red line denoted FC ≥ 0.3 while green line denoted FC < 0.3. (**D**–**F**) Changes of FCs between M1-L and the other 9 nodes pre- and post-sedation in each group. 7 of 9, 1of 9 and 0 of 9 FCs decreased significantly (p[FDR] < .05) after sedation in pre-M1 (**D**) post-M1 (**E**) and control (**F**) group respectively (** *P* <.01, **** *P* <.0001).

Compared with pre-sedation, 27 of 45 FCs within motor areas decreased significantly after sedation in pre-M1 group (p[FDR] < .05, Figure 3D). However, none of these FCs decreased significantly in post-M1 group and control group (Figure 3E, 3F). Compared with pre-sedation, FCs of the ipsilesional M1 with the M1-H, PMd, SMA, Cere-L, Cere-H, Den-L and Den-H were all significantly decreased after sedation in pre-M1 group (p[FDR] < .05, Figure 4D); significantly decreased FC was observed only between M1-L and Den-L in post-M1 group (p[FDR] < .05, Figure 4E); none of the FCs decreased significantly in control group (Figure 4F).

**Figure 5:**
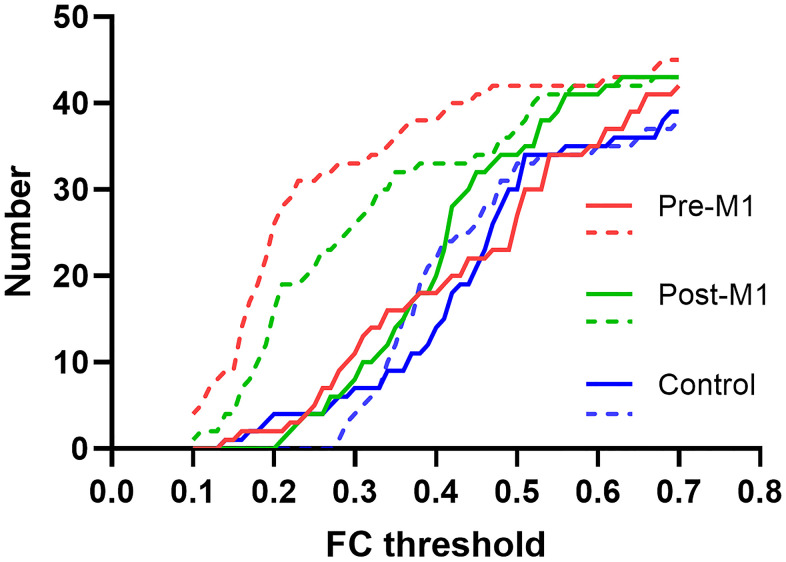
**FC threshold and the corresponding individual FC numbers in pre-M1, post-M1 and control groups.** The solid and dashed line indicated FC before and after sedation separately.

We set FC threshold at 0.3 where maximal differences were observed among the three groups (Figure 5). 34 of 45, 26 of 45 and 4 of 45 FCs were under threshold after sedation in pre-M1, post-M1, and control group respectively (Figure 4A–4C).

## DISCUSSION

The present study used fMRI to evaluate the effect of dexmedetomidine on these patients’ motor network and motor function. We found the LI-V and LI-M at M1 significantly increased in task-fMRI and FCs of motor network significantly decreased after sedation only in the pre-M1 group, rather than in post-M1 and control group.

A certain proportion of patients with tumors involving the motor cortex can maintain normal motor function or mild motor dysfunction even after tumor resection, which is due to cortical compensation [21]. However, the compensatory pathways of the motor network in patients with brain tumors are still under investigation [21–23]. Compensation can take place either in adjacent or in remote brain areas after injury [15]. The motor plasticity is dynamic rather than static, and it can be rapidly changed by external factors such as surgical resection, sedation drugs, and TMS. Duffau et al. reported that the compensatory areas could shift during resection of motor area gliomas, which was named “acute functional reorganization” [24]. Several previous studies have found that the sedative drugs can temporarily reduce the motor capacity of patients with motor area tumors [1–3, 25]. However, its mechanism is still unknown. These findings suggest that in contrast to the intrinsic motor plasticity, the reorganized motor areas and functional connections were more easily to be affected by sedative agents. In this study, we compared the LI of task-fMRI and FCs of rs-fMRI before and after sedation using fMRI. In the task-fMRI, we found that the LI of pre-M1 group rised after sedation, which is consistent with our previous study [1]. The unstabled new connections were temporarily suppressed by sedation, therefore hindering the compensation circuit.

The motor function is tightly interwoven with the correlations within the motor network [16, 21, 26, 27]. In symptomatic patients, disrupted motor network or reduced FCs were found to correlate with worse postoperative outcomes [17]. The pattern of motor network alternation in asymptomatic patients remains controversial. Otten et al. reported a similar motor network between asymptomatic patients and healthy controls [17]. Niu et al. found reduced FCs between the bilateral M1 [10]. In our study, there was little difference in the distribution of functional connectivity strength of all edges within motor network among pre-M1, post-M1 and control groups before sedation. The new connections have been established to compensate for impaired pathways connecting vital nodes after the occurrence of tumor, which helps maintain the motor function. However, the newly developed functional connections don’t completely have the same characters as the intrinsic ones. We found the distribution of connection strength was significantly weakened after sedation in the pre-M1 group. Therefore, dexmedetomidine can unmask or exaggerate the disruption of motor network for such patients.

Cucchiara suggested that the reorganization and compensative pathways only take effect in a completely awake state and were more sensitive to sedation [25]. Dexmedetomidine is associated with a significant drop in the capacity for efficient information transmission [6]. Although the sensory-motor network was reported not suppressed by sedatives in healthy volunteers [5], we have found distinctive results among three groups with brain tumors. Motor network was not inhibited by dexmedetomidine in post-M1 and control groups, but was significantly inhibited in pre-M1 group. Therefore, the compensatory pathways of the motor network, at least part of, were inhibited by sedation. The maximal grip strength was not affected after sedation in most patients, which may be due to dexmedetomidine’s mild sedation effect [1].

The current study also showed the motor network’s stability was closely related with the location of gliomas. In post-M1 and control group, the FCs of ipsilesional M1 and other nodes were stable and not inhibited by sedation. Although in post-M1 group the M1 was invaded by glioma, the motor function was well compensated and was not easily inhibited. In contrast, in pre-M1 group, the FCs of ipsilesional M1 and contralesional M1, contralesional PMd, SMA, and SCb significantly decreased after sedation. That suggested deep involvement of PMd by glioma in pre-M1 group could lead to obvious instability of the motor network. Our findings complied with the previous reports regarding topographical surgical risk for glioma, and also verified the contralesional M1 and bilateral PMA connections are the most important compensatory areas and pathways [28, 29]. fMRI combined with sedation may be a new way to evaluate the vulnerability of the core nodes of the motor network against surgical resection.

The a2-adrenergic agonist or antagonists could enhance or decrease the plasticity in human motor cortex [30, 31]. Dexmedetomidine is a highly selective agonist of α2-adrenoceptor [7, 32]. By reducing the activity of the norepinephrine pathway, dexmedetomidine is often used for sedation, hypnosis, anti-anxiety and analgesia [7, 32]. Dexmedetomidine may decrease the receptor sensitivity or synaptic connections which is upregulated or newly generated in the motor cortex of pre-M1 group. Another possible reason is that dexmedetomidine can reduce BOLD response in the sensory-motor areas [9, 24]. The reorganized motor cortex of pre-M1 group is more sensitive to changes of regional metabolism than normal one. In this study, the inhibition effect of dexmedetomidine on the motor network depends largely on the extent to which M1 was invaded by tumors. Therefore, the motor plasticity significantly changed only in pre-M1 group rather than the other two groups.

Our results will be conducive to enhancing the functional connection of the brain motor network and promoting the recovery of brain motor function through electrical or magnetic stimulation of unstable compensation regions. The small number of patients and the lack of neuroelectrophysiological evidence are the shortcomings of this study. In future research, we will increase the number of patients and confirm these results by intraoperative neuroelectrophysiology.

## CONCLUSIONS

In patients with eloquent area gliomas, dexmedetomidine can inhibit the unstable compensative motor plasticity on both task- and rs-fMRI. This explains why these patients may experience transient limb weakness during sedation. fMRI may be a promising method for elucidating the effect of sedative agents on motor plasticity and the pattern of motor reorganization in these patients.

## Supplementary Material

Supplementary Materials
