# Supplementary material for: Dexmedetomidine inhibits unstable motor network in patients with primary motor area gliomas
Source: Aging (Albany NY). 2021 May 25;13(11):15139–50. doi: 10.18632/aging.203077 (PMC8221338; doi:10.18632/aging.203077)
Supplement: Supplementary Materials [file aging-13-203077-s001.pdf]

SUPPLEMENTARY MATERIALS

Surgery strategy and clinical results

The 21 patients were aged 21-64 years old (mean age 42±12). Sixteen patients received surgical treatments. All the surgeries were performed by Professor S. L. The operation was planned according to the preoperative fMRI examination. During all surgical procedures, ultrasound and intraoperative neurophysiological monitoring were performed to maximize resection and retain function. SSEP and MEP was performed using Cadwell Cascade neurophysiologic monitoring system (Cascade, Cadwell Laboratories Inc, WA, USA). The tumor of eloquent region was resected from the center, and then extended to peripheral tissue gradually. When the interface of resection was suspiciously close to the motor cortex or subcortical tract, a test using mono-polar electrode was placed on the tissue. If the threshold of direct electrical stimulation was 5~6mA, the tissue was thought to be or too close to the eloquent cortex and the risk of post-operative impaired motor functions was considered high, and the resection would be stopped [1]. The ultrasound was performed to check the location and residual nidus of tumor tissue. After operation, the motor deficit of upper limb was only observed in 5 of 6 patients of M1 group. The follow-up time ranged from 1 to 10 months (mean, 6.8 months). At the last follow-up examination, 1 patient experienced tumor relapse. No patient died because of tumor relapse. The motor function was found improved in 2 of 5 patients who suffered postoperative motor deficit.

REFERENCE

1. Ren XH, Yang XC, Huang W, Yang KY, Liu L, Qiao H, Guo LJ, Cui Y, Lin S. [The application of cortical and subcortical stimulation threshold in identifying the motor pathway and guiding the resection of gliomas in the functional areas]. Zhonghua Yi Xue Za Zhi. 2018; 98:653–57. https://doi.org/10.3760/cma.j.issn.0376-2491.2018.09.006 PMID:29534398
